# Analysis of the effects of stromal cells on the migration of lymphocytes into and through inflamed tissue using 3-D culture models^☆^

**DOI:** 10.1016/j.jim.2013.10.004

**Published:** 2013-12-31

**Authors:** Hannah C. Jeffery, Christopher D. Buckley, Paul Moss, G. Ed. Rainger, Gerard B. Nash, Helen M. McGettrick

**Affiliations:** a School of Clinical and Experimental Medicine, College of Medical and Dental Sciences, University of Birmingham, Birmingham, B15 2TT, UK; b School of Immunity and Infection, College of Medical and Dental Sciences, University of Birmingham, Birmingham, B15 2TT, UK; c School of Cancer Sciences, College of Medical and Dental Sciences, University of Birmingham, Birmingham, B15 2TT, UK

**Keywords:** Endothelial cells, Leukocytes, Adhesion, Migration, Inflammation, Fibroblasts

## Abstract

Stromal cells may regulate the recruitment and behaviour of leukocytes during an inflammatory response, potentially through interaction with the endothelial cells (EC) and the leukocytes themselves. Here we describe new in vitro methodologies to characterise the effects of stromal cells on the migration of lymphocytes through endothelium and its underlying matrix. Three-dimensional tissue-like constructs were created in which EC were cultured above a stromal layer incorporating fibroblasts either as a monolayer on a porous filter or dispersed within a matrix of collagen type 1. A major advantage of these constructs is that they enable each step in leukocyte migration to be analysed in sequence (migration through EC and then stroma), as would occur in vivo. Migrated cells can also be retrieved from the constructs to identify which subsets traffic more effectively and how their functional responses evolve during migration. We found that culture of EC with dermal fibroblasts promoted lymphocyte transendothelial migration but not onward transit through matrix. A critical factor influencing the effect of fibroblasts on recruitment proved to be their proximity to the EC, with direct contact tending to disrupt migration. Comparison of the different approaches indicates that choice of an appropriate 3-D model enables the steps in lymphocyte entry into tissue to be studied in sequence, the regulatory mechanism to be dissected, and the effects of changes in stroma to be investigated.

## Highlights

• New models of tissue using endothelial cells, fibroblasts and collagen matrix
• Imaging of T-cell migration through tissue-like constructs
• Fibroblasts promote T-cell migration through endothelial cells.
• Fibroblasts contract collagen gel, restricting T-cell entry.
• In vitro tools to test agents with the potential to alter leukocyte recruitment

## 1 Introduction

During inflammation circulating leukocytes are recruited by blood vascular endothelium (EC), and migrate into the tissue where they fulfil their function in the destruction of invading pathogens and remodelling of damaged tissue. Once the trigger has been eliminated, recruited cells must be cleared to allow resolution. Uncontrolled or ineffective recruitment may be pathogenic, and thus mechanisms controlling these processes have been widely studied. Historically, leukocyte recruitment has been studied using intravital microscopy in animal models, or by in vitro modelling using isolated leukocytes and cultured EC. Based on these studies, paradigms for entry across EC, based on specific adhesion molecules, chemokines and lipids (so-called address codes), have been developed for T-cells and neutrophils (e.g. reviewed by Springer, 1995; Ley et al., 2007). In the case of lymphocytes, capture from flow by cytokine-activated EC is mainly mediated via α_4_β_1_-integrin binding to endothelial VCAM-1, with α_L_β_2_-integrin binding to ICAM-1 supporting transmigration (Luscinskas et al., 1995; McGettrick et al., 2009b). Signals from chemokines (which may vary depending on the inflammatory stimulus) activate the integrins to stabilise the initial interactions (e.g. McGettrick et al., 2009b; Piali et al., 1998), while a downstream signal from prostaglandin D_2_ promotes efficient transendothelial migration (Ahmed et al., 2011).

Less is known about the mechanisms regulating onward migration of leukocytes into tissue and their subsequent behaviour. Intravital and in vitro studies have indicated that T-cells and neutrophils receive signals during transendothelial migration, causing subsequent migratory behaviour and use of adhesion molecules to be modified (Smith et al., 1988; Dangerfield et al., 2002; Burton et al., 2011; Ahmed et al., 2011). Nevertheless, in vitro, lymphocytes appear reluctant to migrate away from the sub-endothelial space into collagen matrix even after hours (Brezinschek et al., 1995; McGettrick et al., 2009a), and they may require additional signals from the tissue stroma to drive efficient penetration (McGettrick et al., 2010). Indeed, it has become increasingly clear that the local stromal environment regulates leukocyte recruitment by endothelial cells (reviewed by McGettrick et al., 2012). For example, we demonstrated that ‘transformed’ tissue stromal cells with characteristics linked to chronic inflammation (e.g., secretory smooth muscle cells or fibroblasts from rheumatoid joints) could potentiate leukocyte recruitment, but that normal fibroblasts could down-regulate recruitment (McGettrick et al., 2009b; Rainger and Nash, 2001).

The above studies have mainly shown the effects of stroma on the initial stages of capture and have revealed less about what happens after, or the mechanisms which control migration through the tissue. This is due, in part, to the lack of amenable 3-dimensional experimental models incorporating EC, stromal cells and interstitial matrix. Since signals received at each stage in the migration process appear to condition leukocytes for the next step, we believe that it is necessary to develop integrated models where leukocytes pass through vascular EC into interstitium containing stromal cells, rather than to study each phase separately, as has been done in much previous work on interaction of leukocytes with stroma (reviewed by McGettrick et al., 2012). Here we describe development of such models. We compared different constructs incorporating human endothelial cell monolayers, gels of collagen type I (the predominant protein of interstitium) and dermal fibroblasts, for their utility in studying lymphocyte behaviour. As expected, fibroblasts modified adhesion to the endothelial monolayer and migration through it, but they could also determine the subsequent efficiency with which lymphocytes penetrated the matrix and influence the rate of onward migration.

## 2 Methods

### 2.1 Isolation of human lymphocytes, fibroblasts and endothelial cells

Venous blood from healthy individuals was collected in EDTA tubes (Sarstedt, Leicester, UK) following informed consent and with approval from the University of Birmingham Local Ethical Review Committee. Peripheral blood lymphocytes (PBL) were isolated by centrifugation on histopaque 1077 followed by panning on culture plastic to remove contaminating monocytes as described (Rainger et al., 2001). Isolated cells were washed, counted using a Cellometer Auto T4 (Peqlab, Southampton, UK), and adjusted to the desired concentration in Medium 199 (M199; Gibco Invitrogen Compounds, Paisley, Scotland) supplemented with 0.15% bovine serum albumin and 35 μg/ml gentamycin (M199BSA; Sigma-Aldrich, Poole, UK).

Tissue samples from the skin were obtained from patients with rheumatoid arthritis (RA) and fibroblasts were isolated as previously described (Salmon et al., 1997). Cells were cultured in RPMI 1640 (Gibco) supplemented with 10% heat inactivated foetal calf serum (FCS), 1 × MEM-non-essential amino acids (100 × stock), 1 mM sodium pyruvate, 2 mM l-glutamine, 100 U/ml penicillin and 100 μg/ml streptomycin (fibroblast medium; all from Sigma) and were used between passages 5 and 9 (McGettrick et al., 2009b).

HUVEC were isolated from umbilical cords using collagenase as previously described (Cooke et al., 1993) and cultured in M199 supplemented with 20% FCS, 1 ng/ml epidermal growth factor, 35 μg/ml gentamycin, 1 μg/ml hydrocortisone (all from Sigma) and 2.5 μg/ml amphotericin B (Gibco) (McGettrick et al., 2009b). All human tissues were obtained with informed consent and with approval from the Human Biomaterial Resource Centre (Birmingham) or NHS Staffordshire Research Ethics Committee.

### 2.2 Co-cultures of endothelial cells and fibroblasts on separate filters

**Fig. 1:**
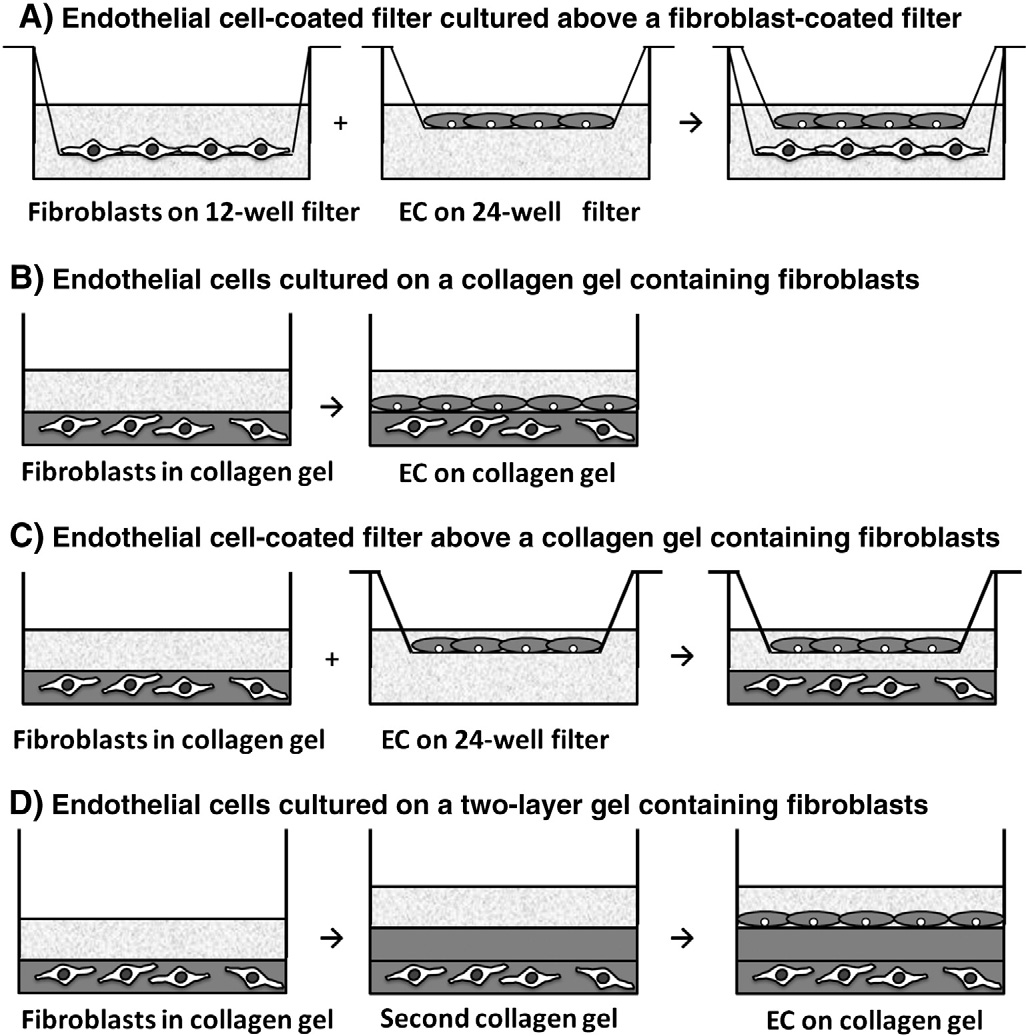
Schematic representation of multi-cellular, multi-layered, 3-D in vitro constructs to examine the process of lymphocyte migration into and through tissue. (A) EC and fibroblasts were cultured inside 24-well and 12-well 3.0 μm pore filters for 24 h respectively. Co-cultures were established by inserting the EC-coated 24-well filter into the fibroblast-coated 12-well filter. (B–D) Fibroblasts were incorporated into a collagen solution prior to the formation of a gel. (B) Co-cultures were established by seeding EC on the surface of the gel or (C) inside 12-well 3.0 μm pore filters for 24 h before the filter was placed above the gel. (D) Alternatively, a second blank layer of collagen gel was overlaid onto the fibroblast containing gel, on top of which EC were seeded.

Fibroblasts and EC were dissociated using trypsin/EDTA (Sigma) and were cultured in fibroblast medium on the inner surfaces of 24-well and 12-well 3 μm pore Transwell filter inserts (BD Pharmingen, Cowley, UK) respectively, as described (McGettrick et al., 2010) (Fig. 1A). Fibroblasts were seeded at 1.5 × 10^5^ cells/filter and HUVEC were seeded at 1.0 × 10^5^ cells/filter to yield confluent monolayers within 24 h. After 24 h, culture media were removed and the 24-well inserts were fitted into the 12-well inserts, with 200 μl fibroblast medium added to the surface of each filter and 1.5 ml to the lower chamber. Cells were co-cultured together for 48 h, with 100 U/ml TNF alpha (R&D Systems, Abingdon, UK) in combination with 10 ng/ml IFN gamma (Peprotech Inc., London, UK) added for the second 24 h when desired. For comparison, parallel cultures of HUVEC or fibroblasts were maintained alone on their original filters.

### 2.3 Co-cultures of endothelial cells and fibroblasts incorporating collagen gels

To form collagen gels, ice-cold rat-tail type 1 collagen dissolved in acetic acid (2.15 mg/ml; First Link Ltd, West Midlands, UK) was mixed with ice cold 10 × concentrated M199 in the ratio 830:170 and the pH was neutralised by addition of ice cold 1 N NaOH. For each 1 ml of gel, 160 μl FCS was added, yielding a final collagen concentration of ~ 1.5 mg/ml. Gels were dispensed into 12-well or 6-well plates (400 μl or 1 ml respectively), allowed to set for 15 min at 37 °C and then equilibrated with fibroblast culture medium for at least 24 h.

When desired, fibroblasts were incorporated into the gel (Fig. 1B–D). Fibroblasts were dissociated as above, counted and adjusted to the desired concentration in the ice cold FCS (5 × 10^4^ cells/64 μl for 12-well or 2 × 10^5^ cells/160 μl for 6-well). FCS/fibroblasts were mixed with neutralised gel solution, 64 μl FCS + 400 μl gel or 160 μl FCS + 1 ml gel, before it was dispensed into 12-well or 6-well plates respectively and allowed to gel as above. For some assays, a layer of empty gel was formed on top of a gel containing fibroblasts (Fig. 1D). In this case, once the lower fibroblast-containing gel had formed, it was overlaid with fresh gel solution (300 μl/12 well) that was set for 50 min at 37 °C.

To form co-cultures, HUVEC were either seeded directly onto the surfaces of the single or double layer gels (Fig. 1B,D), or inside of a 12-well 3 μm pore Transwell filter which was placed above the gel (Fig. 1C). Co-cultures were maintained in fibroblast medium for 48 h, with 100 U/ml TNF + 10 ng/ml IFN added for the second 24 h when desired.

Several simplified models were set up for comparison when studying lymphocyte adhesion and migration: parallel cultures of HUVEC were made on or over ‘empty’ gels; fibroblasts were maintained in gels without added HUVEC or gels were maintained empty. In the last case, we also studied gels made at higher collagen concentrations by starting with rat-tail type 1 collagen dissolved in acetic acid at 9.18 mg/ml (Becton Dickinson, Oxford, UK) and pre-diluting this as desired with acetic acid before formation of gels as above.

### 2.4 Adhesion and migration of lymphocytes on endothelial cells and fibroblasts on separate filters

Immediately before assay, medium in the lower chamber and between the filters was replaced with M199BSA (see Fig. 1A). Purified PBL (200 μl at 2 × 10^6^ cells/ml) were added to the upper 24-well filter. PBL were allowed to settle and adhere at 37 °C in a CO_2_ incubator for 10 min, after which non-adherent PBL in the 24-well filter were collected by washing the filter twice (fraction A). Fresh medium was added to the upper well and after 24 h, migration was stopped by transferring each filter into a fresh well, leaving the cells which had migrated through both filters in the original lower chamber (fraction B). Cells which had migrated through the first filter but were not adherent to the lower 12-well filter were collected by washing the filter twice (fraction C). The two filters were treated with Accutase (Gibco) to dissociate the cells associated with the endothelial monolayer or fibroblast monolayer, and these were collected by washing the filters twice (fractions D or E respectively).

For comparison, the same number of PBL were added to endothelial cells or fibroblasts cultured alone on their respective filters. The equivalents of fractions A, B and D or E were collected.

The cells in the fractions were counted using flow cytometry; when desired we also analysed the proportion of the major subpopulations of T cells in the different fractions (see below for detail). Total adherent cells were calculated by subtracting fraction A from the total added. Transendothelial migration was quantified as the sum of the PBL located in the compartments beneath the endothelial monolayer (fractions B, C and E; i.e. in the lower chamber, in the medium in between the two filters, and attached to the fibroblasts). Migration through the fibroblasts was determined from the number of cells in the lower chamber (fraction B). The numbers in the different categories were normalised as follows: adhesion as percentage of all cells added; transendothelial migration as % of total adherent; trans-fibroblast migration as percentage of those delivered to the fibroblasts.

### 2.5 Adhesion and migration of lymphocytes on co-cultures incorporating collagen gels

PBL (2 × 10^6^ cells/ml; 2 ml for a six-well; 1 ml for a twelve-well) were added and allowed to settle on HUVEC cultured on gels containing fibroblasts (Fig. 1B,D) and incubated at 37 °C in a CO_2_ incubator for the desired time. Non-adherent cells were then removed by gentle washing of the surface with M199BSA. The endothelial surface was observed using a phase-contrast microscope with a motorised focus and digital camera under computer control using Image-Pro Plus software (DataCell Ltd, Finchampstead, UK). Digitised z-stack images were acquired at 2 μm intervals through the depth of the gel in five random fields and analysed offline using the same software. The numbers of PBL were counted as they came into and out of focus during playback, averaged over the fields and then converted to cells per mm^2^ using the calibrated microscope field dimension. The number was multiplied by the known surface area of the HUVEC monolayer to calculate the total number adherent. This number was divided by the known total number of PBL added, to obtain the percentage of the PBL that had adhered. At the endothelial layer, the PBL count was divided into those which were phase bright (above EC; fraction X) and those which were phase dark (migrated just below EC; fraction Y). From these counts and the sum of PBL further into the gel (fraction Z), the percentages of adherent PBL that had undergone transendothelial migration ((Y + Z) / (X + Y + Z)) × 100% and the percentage of migrated cells that had penetrated into the collagen gel (Z / (Y + Z)) × 100% were calculated.

The vertical position of those cells within the gels was also recorded. This was done by counting PBL in 18 μm ‘slices’ made up from 5 consecutive images (starting *after* the image of the endothelial monolayer referred to above), and assigning them a depth equal to the midpoint of that slice. The average depth of penetration was calculated by multiplying the midpoint depth by the number of cells found within that slice (averaged for the 5 fields), summing these values, and dividing the sum by the total number of cells in the stack. The total gel thickness was also measured (from endothelial layer to base of dish), and the proportion of PBL within the upper and lower halves of the gel was also calculated. In addition, fibroblasts in the gel were counted and depth assigned in a similar manner; these large extended cells could appear in multiple images, and their nucleus was used to assign location.

Several variants on this procedure were used for comparison. PBL were added to HUVEC on ‘empty’ gels, or added to gels which contained fibroblasts but did not have an endothelial layer, or added to empty gels. Incubation and analysis of numbers and position of cells were carried out essentially as before. Percentage of PBL entering the gels in the latter cases (without a HUVEC layer) were calculated from the total number added to the top of the gel or relative to the blank gel control.

In separate assays, with HUVEC cultured on filters above gels (Fig. 1C), PBL were added to the filter and incubated as above for 24 h. At that time, non-adherent cells were washed from the filter and counted, the filter was removed, and the gel was then analysed as above for the number and position of PBL on or in it. In some cases, the culture was then returned to the incubator, and position of PBL re-evaluated after a further 20 h.

### 2.6 Retrieval of cells from the collagen gel for analysis of phenotype

At the end of the imaging of gels, constructs in which endothelial cells were cultured on the surface of the gel were treated with dispase II (1 mg/ml; Sigma) for 15 min to dissociate the endothelial monolayer and lymphocytes associated with it. After microscopic check of dissociation, the cells were collected using two washes with M199BSA. For gels without endothelial monolayers, non-adherent cells were collected from the top by two similar washes. The gels were then digested in 1 ml of 2 mg/ml collagenase III (Sigma) for 30 min at 37 °C in a CO_2_ incubator, cells collected and washed in PBSA. The cells retrieved from the surface and from the gel were analysed for their content of lymphocyte subsets by flow cytometry (see below).

### 2.7 Flow cytometry

Freshly isolated, non-adherent or the various migrated cells were labelled with a combination of the following antibodies: anti-CD4-PE, anti-CD8-FITC, anti-CD3-PerCP; anti-CD62L-FITC, CD45RA-PE (all from Becton Dickinson, Oxford, UK), anti-CD45RA-CY5 (Serotech, Oxford, UK), anti-CD4-efluor405; anti-CD8a-efluor605 and anti-CD19-PE-Cy7 (all from eBiosciences, Hatfield, UK) for 30 min on ice. Labelled cells were spiked with a known volume of Flow-Count Fluorospheres (Beckman Coulter, High Wycombe, UK). Cells were counted and their fluorescence analysed using a Cyan flow cytometer and Summit software (both from Dako). In some cases, cells were enumerated by passing the entire sample through the flow cytometer. In this way, we could separately count and calculate the percentages of the following subsets that adhered, transmigrated or penetrated into the gels: CD4^+^ or CD8^+^ T-cells (CD3^+^), which were of naive (CD45RA +, CD62L +), effector memory (CD45RA −, CD62L −) or central memory (CD45RA −, CD62L +) phenotypes; CD19^+^ B-cells (Supplemental Fig. 1).

Endothelial cells were incubated with non-conjugated antibodies against E-selectin (1.2B6) or VCAM-1 (1.4C3; both Dako, Ely, UK) for 30 min at 4 °C, washed and incubated with goat anti-mouse FITC-conjugated secondary antibody (Dako) for 30 min at 4 °C as previously described (McGettrick et al., 2009b, 2010). Fibroblasts were incubated with APC-conjugated anti-ICAM-1 (BD Pharmingen, UK) for 20 min at 4 °C. Subsequently, cells were washed and incubated with enzyme-free cell dissociation buffer (Gibco) for 30 min. The dissociated cells were analysed by flow cytometry and data were expressed as median fluorescent intensity (MFI).

### 2.8 Gene expression

Endothelial mRNA was isolated using the RNeasy Mini Kit (Qiagen, Crawley, UK). Gene expression of the chemokines CXCL9, -10, and -11 was analysed by reverse transcription (RT) PCR, followed by densitometry of product bands run on agarose gel containing ethidium bromide, as described (McGettrick et al., 2009b, 2010). Data were expressed as a percentage of the β-actin bands.

### 2.9 Statistical analysis

Variation between multiple treatments was evaluated using analysis of variance (ANOVA), followed by comparison of treatments by Bonferroni (inter-treatment) or Dunnett (comparison to control) test as appropriate. Effects of single treatments were analysed by paired or unpaired t-test as appropriate. P < 0.05 was considered as statistically significant.

## 3 Results

### 3.1 Effects of co-culture on migration of PBL through EC and fibroblasts on separate filters

**Fig. 2:**
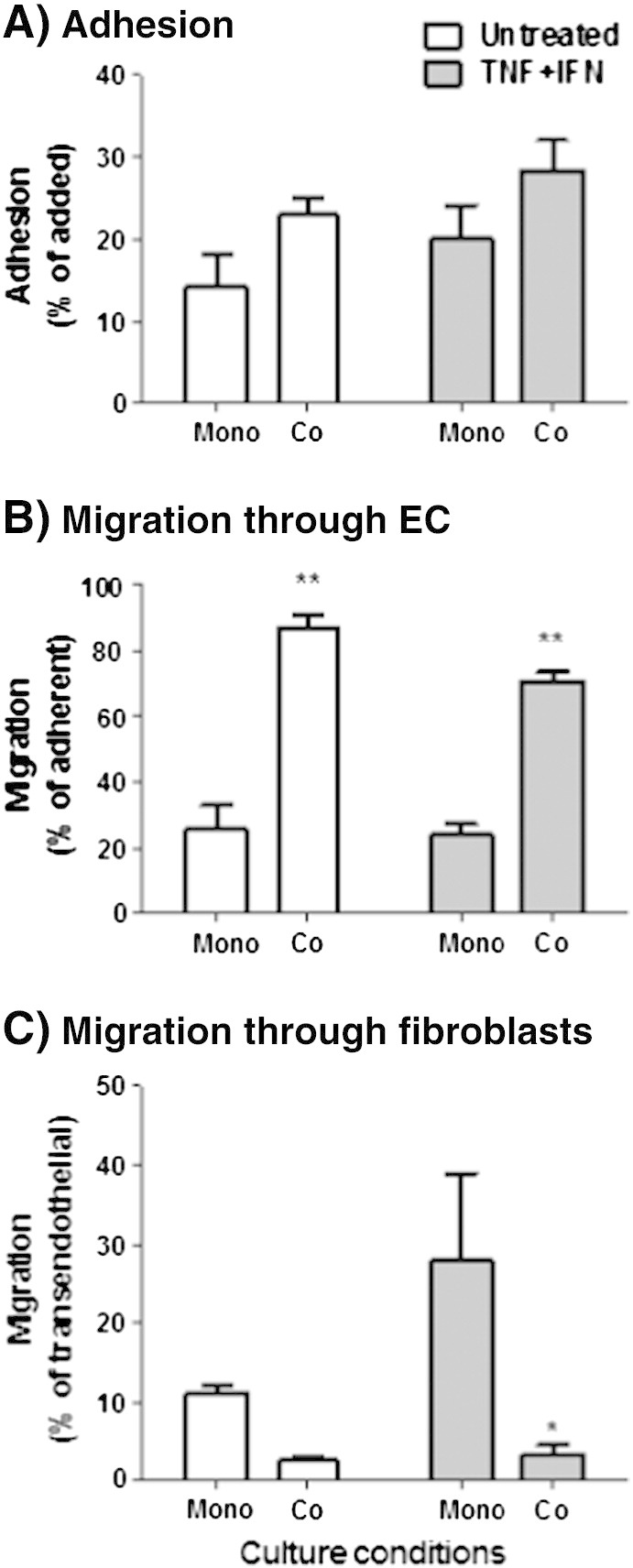
Adhesion and migration of lymphocytes through co-cultures on filters. Co-cultures were established by inserting an EC-coated 24-well filter into a fibroblast-coated 12-well filter for 24 h prior to cytokine treatment. EC (none) and fibroblast monolayers were used as controls. PBL were settled for 10 min after which non-adherent cells were washed off. PBL adhesion (A) migration through EC (B) and fibroblasts (C) were assessed at 24 h using flow cytometry. Data were expressed as a percentage of cells added (A) or percentage of adherent cells (B–C). In the case of migration through fibroblasts, data were normalised for number of cells that were adherent to fibroblasts in the co-cultures. ANOVA shows a significant effect of fibroblasts on PBL migration through EC (p < 0.001) and through fibroblasts (p < 0.05). Data are the mean ± SEM from 4 to 6 independent experiments. * = p < 0.05 and ** = p < 0.01 compared to mono-cultures of EC or fibroblasts for matched cytokine treatment by Bonferroni post test.

The level of adhesion to EC was slightly higher for cytokine treated than unstimulated cultures, and co-culture with fibroblasts tended to increase this level, but neither effect was statistically significant (Fig. 2A). The migration of the adherent cells through the EC was again similar whether cytokines had been added to the culture or not, but in either case, co-culture with dermal fibroblasts greatly augmented the transendothelial migration (Fig. 2B). These trends are similar to those we previously reported with the two filter model (McGettrick et al., 2010). Lymphocytes are known to take longer to migrate across these artificial filters (hours) than to transit through the EC (McGettrick et al., 2009a). Endothelial cells in these filter models are able to respond to cytokine treatment as expected, up-regulating surface expression of the adhesion receptors, E-selectin and VCAM-1 and producing chemokines, including CXCR3 ligands at the mRNA level (Supplemental Fig. 2). A combination of prolonged settling periods and non-specific delays in transit across the filters are likely to explain the similarities in migration observed between cultures treated with or without cytokines. We also analysed onward migration through the layer of fibroblasts. When fibroblasts were cultured alone, lymphocytes migrated through the monolayer quite readily, with a tendency for more to migrate when the fibroblasts have been treated with cytokines (Fig. 2C). Interestingly, PBL migrated across fibroblasts in the co-cultures much less efficiently than in the mono-cultures (Fig. 2C).

### 3.2 Effects of fibroblasts on migration of PBL through EC and directly into collagen gels

**Fig. 3:**
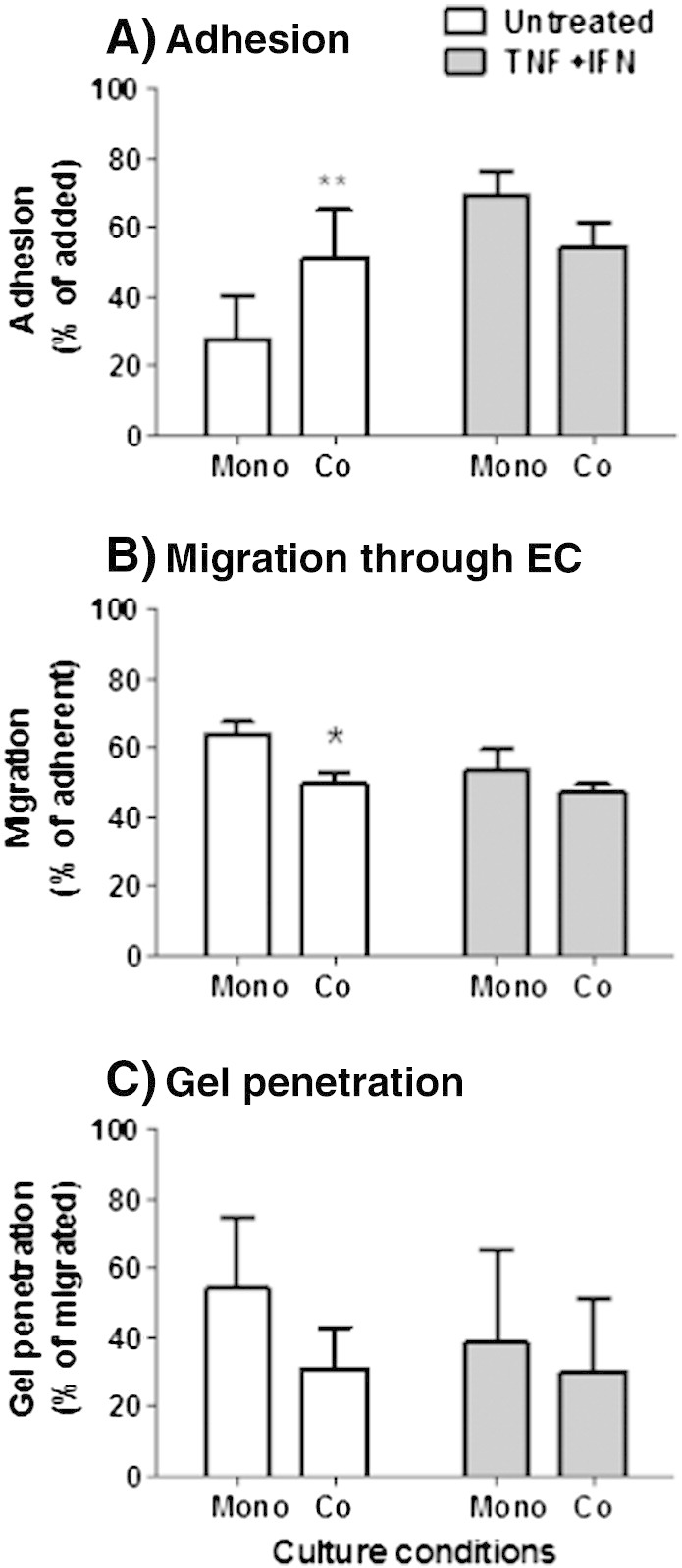
Migration of lymphocytes through EC and into gels containing fibroblasts. Fibroblasts were incorporated into a gel on top of which EC were cultured for 24 h, prior to treatment with (filled bars) or without (open bars) TNF + IFN. PBL were allowed to adhere for 3 h on untreated EC or for 10 min on cytokine-treated cultures, after which non-adherent cells were washed off. PBL adhesion to EC (A) was assessed immediately following removal of non-adherent cells. Subsequently, PBL migration (B) and penetration of the underlying collagen gel (C) were assessed at 24 h after the initial addition of cells. Data were calculated as the percentage of the adherent cells (A–B) or the percentage of migrated PBL, which had entered the gel (C). Data are mean ± SEM from 4 to 5 independent experiments. * = p < 0.05 and ** = p < 0.01 compared to EC cultured alone (none) by paired t-test.

The above results raised the question whether fibroblasts would similarly increase the migration of PBL through endothelial cells when presented in a 3-D matrix, and/or influence progress of PBL through that matrix. To test this, we designed a construct in which we could visualise PBL migration through and away from the EC, and then through a collagen gel incorporating fibroblasts (Fig. 1B). For unstimulated cultures, PBL were allowed to settle for 3 h on the EC to allow adequate levels of adhesion for migration analysis. Under these conditions, fibroblasts promoted PBL adhesion, but significantly reduced the efficiency of subsequent transendothelial migration of the adherent cells, and also tended to inhibit the entry of those cells that had crossed the endothelium into the gel (Fig. 3; clear bars). After treatment with cytokines, only 10 min settling was needed to obtain efficient adhesion to EC (as previously described; McGettrick et al., 2009a). However, in this case fibroblasts had little effect on the ability of PBL to adhere (Fig. 3A; filled bars). They retained a tendency to reduce migration through the endothelium and penetration into the underlying gel (Fig. 3B–C; filled bars), but neither effect was statistically significant. Thus in this model, fibroblasts failed to promote transendothelial migration as seen in the filter model, but did retain a tendency to hinder migration of cells after crossing that barrier.

### 3.3 Effects of fibroblasts on migration of PBL through EC on filters and then into collagen gels

In some co-cultures on gels, we observed that the endothelial monolayer retracted and/or some cells detached during the culture (Supplemental Fig. 3). While these cultures were not used for assay, this does suggest that the close proximity of fibroblasts to EC may alter their barrier function. Indeed, we have previously reported that culture of EC and fibroblasts inhibited the recruitment of PBL when they were in close contact on opposite sides of 3.0 μm pore filters, but not when 0.4 μm pore filters were used (McGettrick et al., 2010). To test how migration into 3-D matrix might be influenced by fibroblasts co-cultured with EC but not in direct contact, we modified the construct so that the EC were cultured on filters above collagen gels incorporating fibroblasts, with the two cell types separated by 600–800 μm (Fig. 1C).

**Fig. 4:**
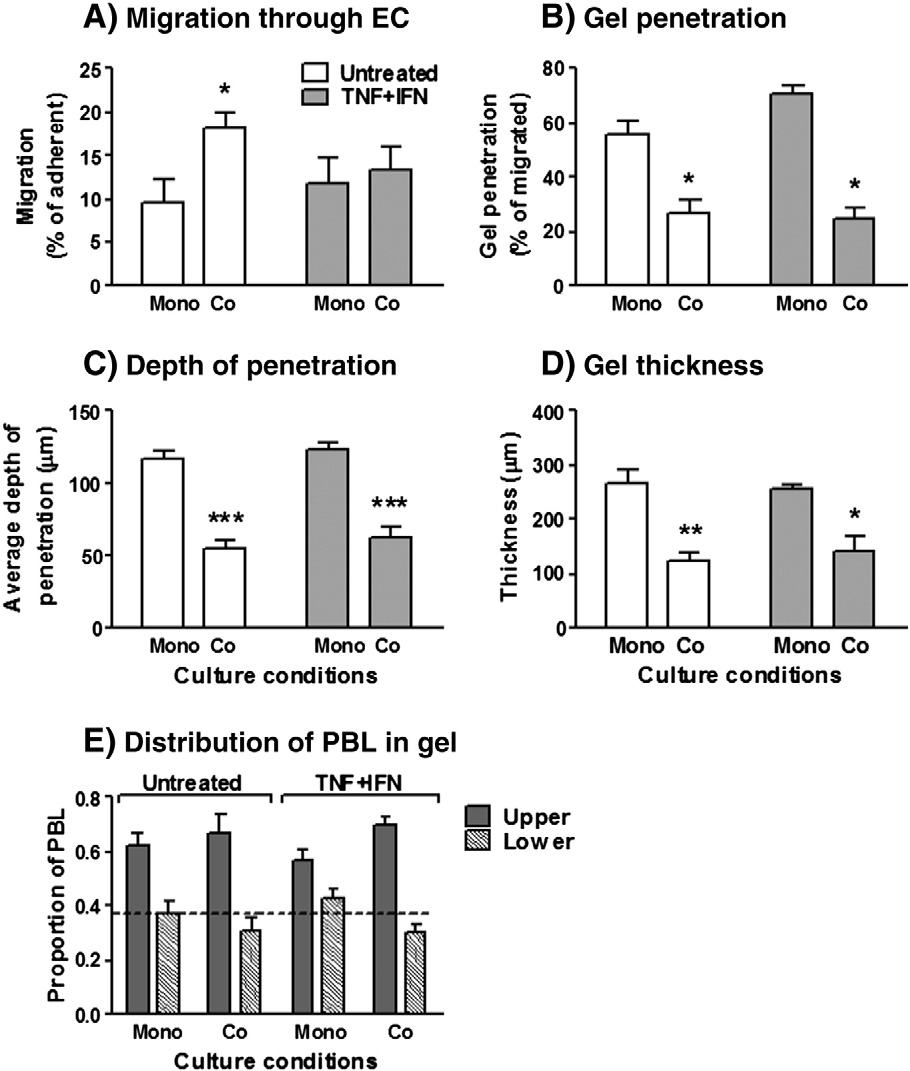
Migration of lymphocytes through EC-coated filters into gels. EC were cultured on filters above gels incorporating fibroblasts. PBL migration (A) and gel penetration (B) were assessed at 44 h by flow cytometry. Data were expressed as percentage migration of those cells that adhered (A) and percentage of migrated PBL which had entered the gel (B). (C) Average depth PBL migrated into the gel, (D) depth of gel and (E) proportion of PBL in the upper and lower halves were measured using commercial image analysis software at 24 h (C). In E, ANOVA shows a significant effect on distribution of PBL in the gel, p < 0.001. Data are mean ± SEM from 3 to 6 experiments. * = p < 0.05, ** = p < 0.01 and *** = p < 0.001 compared to EC (none) for matched cytokine treatment by paired t-test or (E) by Bonferroni post test.

In this construct, we observed similar adhesion of PBL to EC for mono- and co-cultures, with or without treatment with cytokines (data not shown). In the absence of cytokines, fibroblasts in the gel markedly increased the migration of PBL through the endothelial layer on the filter compared to EC cultured alone, but this effect was much reduced when cultures had been treated with cytokines (Fig. 4A). Interestingly, however, fibroblasts significantly reduced the entry of the migrated PBL into the collagen gel, both in the untreated and cytokine-treated cultures (Fig. 4B). Of note, fibroblasts cultured within gels respond appropriately to cytokine-stimulation, up-regulating ICAM-1 expression and secretion of CXCL1 and CXCL10 to a similar level as that observed by fibroblasts cultured on plastic (i.e. in the absence of collagen) (Supplemental Fig. 4). Moreover, these responses were maintained during co-culture with endothelium (Supplemental Fig. 4). Thus fibroblasts are capable of responding to cytokines and also suppressing T-cell entry into the gel, indicating a role for other factors in this effect. Thus, so far, fibroblasts tended to promote the migration of PBL across EC when direct contact was prohibited, but tended to inhibit onward migration in co-culture.

To gain insight into the latter effect, we examined the distribution of PBL and fibroblasts within the gels. The distances PBL migrated into the gels after 24 h were significantly reduced in the presence of fibroblasts for unstimulated or cytokine-treated cultures (Fig. 4C). Similar reduction in depth was also observed at 44 h (data not shown). However, in examining the position of fibroblasts in the gel, we found that the depth of the gels was much less in their presence than in their absence (Fig. 4D). While we observed that fibroblasts were evenly distributed through the depth of the gel (data not shown), they had significantly contracted the gel. To evaluate the depth of penetration by PBL in a manner independent of gel depth, we calculated the proportions of PBL in the upper and lower halves of the gel. On average there were significantly more PBL in the upper half of the gel compared to the lower half (ratio about 60:40) (Fig. 4E). In addition, the proportion in the upper half was slightly higher (and the proportion in the lower half slightly lower) when fibroblasts were present in the gel. Thus, fibroblasts could again inhibit the proportion of cells crossing EC that could enter a gel, and had a small effect on the distance travelled by those that did enter the gel, but this effect might be attributable to contraction of the gel.

### 3.4 Effect of fibroblasts on migration of PBL into collagen gels in the absence of EC

To investigate the apparent barrier function of fibroblasts further, we tested migration into gels containing fibroblasts but without an endothelial monolayer in the absence of cytokine treatment. Again, fibroblasts significantly reduced the thickness of the gel compared to a gel containing no fibroblasts (depth = 110 ± 10 μm vs 249 ± 24 μm respectively; mean ± SEM, n = 5–6; p < 0.001 by unpaired t-test). When PBL were settled on the gel for 24 h, we observed similar numbers of PBL entered gels containing fibroblasts (17 ± 6% vs 19 ± 2% of added cells migrated into gels with or without fibroblasts respectively; mean ± SEM, n = 5), however the cells travelled shorter distances into the construct (59 ± 9 μm vs 123 ± 5 μm; mean ± SEM, n = 5–6; p < 0.001 by unpaired t-test) when compared to the empty gel. Again significantly more PBL were observed in the upper half of the gel compared to the lower half (ratio about 60:40) (p < 0.01 by paired t-test; data not shown). In contrast to the endothelial-gel model, fibroblasts had no effect on the proportion of PBL in the upper (or lower) half of the gel when compared to gels containing no fibroblasts (data not shown).

**Fig. 5:**
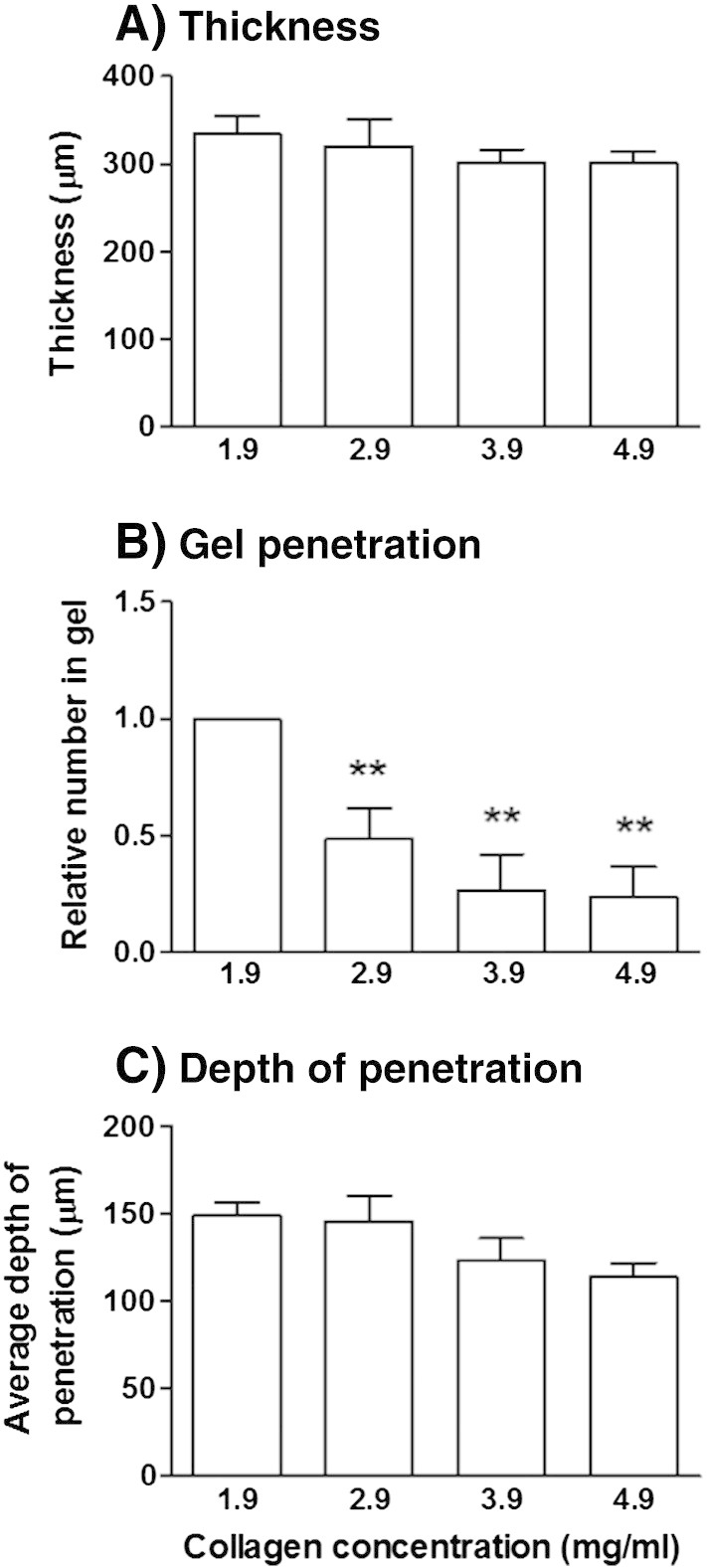
Migration of lymphocytes into collagen gels of differing concentrations. Gels were formed (1.9–4.9 mg/ml) in the absence of fibroblasts and PBL were allowed to migrate for 24 h. (A) Depth of the gel. (B) Number of PBL entering each gel, relative to the standard concentration gel (1.9 mg/ml), was calculated and (C) the mean depth to which the cells penetrated was determined. In B, ANOVA shows a significant effect of gel concentration on the ability of PBL to penetrate the gel, p < 0.001. Data are mean ± SEM from 4 experiments. ** = p < 0.01 compared to 1.9 mg/ml collagen gel by Bonferroni post test.

In vivo, the thickness of collagen bundles and pore size (gaps between individual bundles) is highly variable, for example, in human skin, pore diameters of 2–10 μm been reported (Wolf et al., 2009). Based on previous reports, we expected the single collagen gels to yield a uniform density of collagen with pore diameters of ~ 2 μm between bundles (Wolf et al., 2009). To test whether the above effects on lymphocyte migration were attributable to the gel contraction rather than effects from interactions with the fibroblasts, we formed gels at higher concentrations, over the range of 1.9–4.9 mg/ml in the absence of fibroblasts and EC. Increasing the collagen concentration had little effect on the overall thickness of the gel formed (Fig. 5A), but did cause significant, progressive reduction in the number of PBL entering the gel (Fig. 5B). Interestingly, for those cells that did enter the gel, depth of penetration was little affected by the gel density (Fig. 5C). Collectively, the above data suggest that the fibroblasts contract the gel to about double its density, and this would be sufficient to inhibit entry of PBL into the gel, but would have little effect on their migration within the gel itself.

### 3.5 Effects of fibroblasts in a double-layered gel on migration of PBL

To try to separate effects of gel contraction and of agents released by fibroblasts, constructs were designed in which fibroblast-containing gels were overlaid with a blank gel (Fig. 1D). Fibroblasts were observed in the lower part of the gel depth, and significantly decreased the overall depth of the double gel from 1206 ± 15 μm to 1075 ± 24 μm (mean ± SEM, n = 3–5; p < 0.01 by unpaired t-test). We observed no difference in the number of PBL that entered gels containing fibroblasts (13 ± 1% vs 14 ± 2% of added cells migrated into gels with or without fibroblasts respectively; mean ± SEM, n = 3–5), indicating little if any modification of the collagen density in the upper blank gel. The depth of penetration of the PBL in the double gel construct was slightly greater in the presence of fibroblasts in the lower gel layer (262 ± 10 μm vs 228 ± 13 μm; mean ± SEM, n = 3–5) but the difference was not statistically significant.

**Fig. 6:**
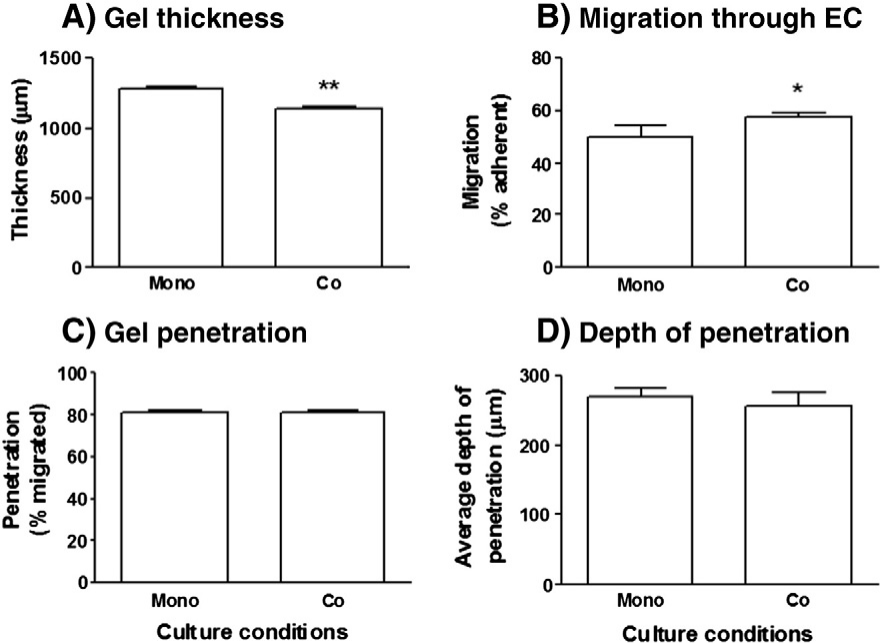
Migration of lymphocytes through EC into a multi-layer gel. EC coat the upper surface of an empty collagen gel on top of a second layer of gel incorporating fibroblasts. (A) Depth of the gel. (B) PBL migration and (C) gel penetration were assessed at 24 h and expressed as percentage migration of those cells that adhered (B) and percentage of migrated PBL which had entered the gel (C). (D) Average depth PBL moved into the gel. Data are mean ± SEM from 6 to 7 experiments. * = p < 0.05 and ** = p < 0.01 compared to EC control (none) by paired t-test.

Since the effects of fibroblasts on PBL migration were reduced when they were remote from the surface, we tested whether this applied when double gels were overlaid with EC. The double gel separated the EC and fibroblasts by about 800 μm and the overall gel thickness was slightly but significantly reduced by the presence of fibroblasts (Fig. 6A). Under these conditions, fibroblasts induced a small but significant increase in PBL transendothelial migration (Fig. 6B), but had no effect on the initial adhesion (data not shown), number of PBL entering the gel, or the depth to which they penetrated (Fig. 6C,D).

Taken together, the above results suggest that fibroblasts can have effects on adhesion to EC and transmigration remotely, but effects on subsequent migration in tissue are dependent on direct contact and/or modification of matrix density.

### 3.6 Migratory behaviour of different lymphocyte subsets

In principle, the effects of fibroblasts noted above might be greater or less for different subsets of the PBL. In that case, studies of mixed populations might yield averaged results which hide or underestimate the specific effects. We thus evaluated separately the behaviours of the main subsets within the PBL, using flow cytometry to identify them in the various collected fractions. We found in the two-filter model that fibroblasts promoted transendothelial migration similarly for CD4 and CD8 subsets of T-cells, and that hold-up of T-cells by fibroblasts after they had migrated through EC was also similar for these subsets (data not shown).

**Fig. 7:**
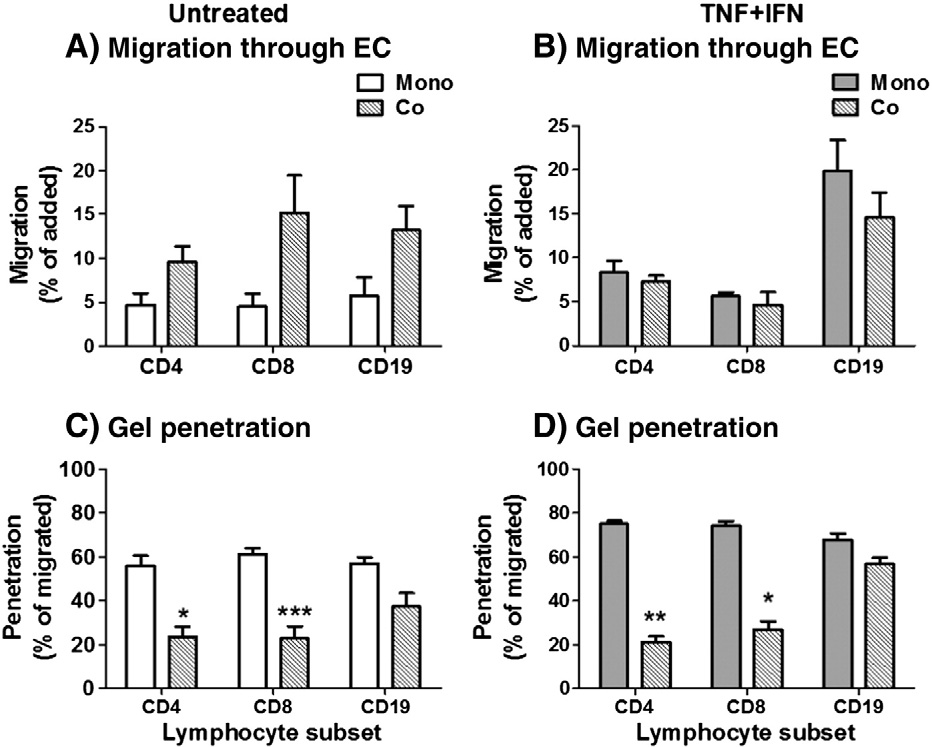
Migration of T and B-cells into collagen gels. PBL were retrieved from the constructs and the behaviours of individual subsets were assessed by flow cytometry at 44 h. Data were expressed as percentage migration of those cells that were added (A&B) and percentage of migrated PBL, which had entered the gel (C&D). Data are mean ± SEM from 3 to 5 experiments. * = p < 0.05, ** = p < 0.01 and *** = p < 0.001 compared to EC (none) for matched PBL subset by paired t-test.

**Fig. 8:**
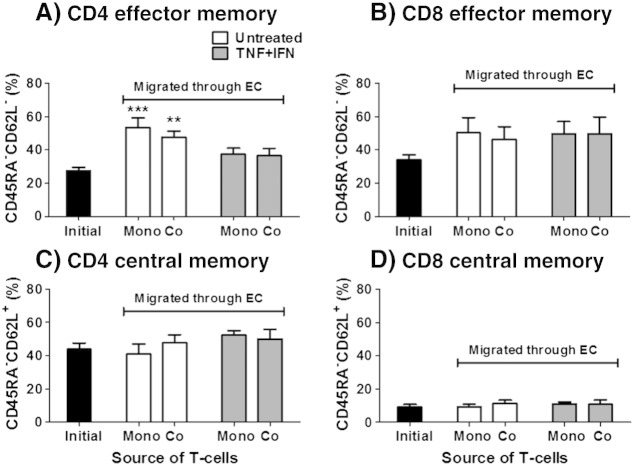
Migration of effector memory T-cells into collagen gels. PBL were retrieved from constructs and the percentage of effector memory (CD45RA^−^CD62L^−^) or central memory (CD45RA^−^CD62L^+^) phenotype in the initial, migrated and gel penetrated populations were determined by flow cytometry at 44 h. Data were expressed as percentage of effector (A&B) or central (C&D) memory CD4 or CD8 T-cells that had migrated through the endothelium. ANOVA shows a significant effect of CD45RA^−^CD62L ^−^phenotype on CD4 T-cell migration (p < 0.001). Data are mean ± SEM from 3 to 6 experiments. * = p < 0.05, ** = p < 0.01 and *** = p < 0.001 compared to initial population by Dunnett post test.

When EC were cultured on filters over gels, we assessed B-cells as well as the CD4 and CD8 populations of T-cells (Supplemental Fig. 1). Migration through the EC in unstimulated co-cultures was higher for all three cell types when compared to mono-cultures (Fig. 7A), while no subset was affected by co-culture in the cytokine stimulated cultures (Fig. 7B). In contrast, while fibroblasts inhibited entry of the CD4 and CD8 T-cells into the underlying gel, B-cells penetrated gels containing fibroblasts nearly as well as empty gels (Fig. 7C,D). Similar observations were made in constructs formed in the absence of endothelial monolayers, where fibroblasts decreased T-cell, but not B-cell, penetration of the gel (data not shown). For the CD4 and CD8 T-cells, we also compared the behaviour of the naïve, effector memory or central memory cells. Overall, memory T-cells preferentially migrated across EC mono- and co-cultures compared to naïve T-cells (data not shown). Interestingly, migration through EC tended to select for effector memory T-cells (Fig. 8A–B), and not central memory T-cells (Fig. 8C–D). Moreover, no further selection was observed when fibroblasts were present or at the level of T-cells entering into the gel (data not shown). Similarly, in the absence of an EC monolayer, migration into the gel also tended to select for effector, rather than central, memory T-cells (data not shown). This indicates that the selection of effector memory cells was not due to the endothelial monolayer but rather the efficiency of individual memory populations.

## 4 Discussion

Stromal cells can regulate the recruitment and behaviour of leukocytes during an inflammatory response through interaction with EC and the leukocytes themselves (reviewed by McGettrick et al., 2012). Here we developed novel 3-D in vitro constructs for studying effects of stromal cells on leukocyte recruitment, especially migration of lymphocytes through endothelium and its underlying matrix. Constructs were built up stepwise, with EC cultured above a stromal layer incorporating fibroblasts, using porous filters and/or a matrix of collagen type 1 (Fig. 1). A major advantage of these constructs is the ability to analyse leukocyte migration through EC and then stroma, with the migrating cells conditioned by each step in order, as would occur in vivo. Retrieval of cells from the different migrated pools is also possible, allowing subset selectivity to be analysed, as well as functional responses of migrated cells in separate assays if desired. Here we evaluated mechanisms regulating migration of different populations of PBL, with or without addition of inflammatory cytokines. We found that in general, culture of EC with dermal fibroblasts promoted transendothelial migration but not transit through matrix. However, results were dependent on the format in which the EC and fibroblasts were presented to each other.

Transwell filters are frequently used in chemotaxis and transendothelial migration assays, though less commonly combined with fibroblasts and gels. In our two-filter model, fibroblasts augmented PBL migration through EC, but transit through the fibroblast layer was actually inhibited for PBL that had crossed the EC compared to those applied directly to fibroblasts. This suggests that fibroblasts may retain transmigrated T-cells, either because transendothelial migration altered the T-cells or because the fibroblast monolayers became less easy to penetrate when cultured with EC. Notably, our previous studies showed that after migration through EC, T-cells passed *more* efficiently through monolayers of lymphatic endothelial cells (Ahmed et al., 2011), indicating that their migratory ability is not impaired. Others have reported that dermal fibroblasts isolated from patients with scleroderma promoted mononuclear leukocyte migration through EC cultured on filters (Denton et al., 1998). In that study the fibroblasts were cultured on the plastic well underneath the filter, so that onward migration could not be examined. While our model provides an opportunity to study the effects of the stroma upon leukocyte migration though the two cell layers independently, the fibroblasts are presented in a layer rather than matrix. Additionally, they lack direct interaction with EC, and so only interactions mediated by soluble factors are modelled. Whether this is the case in vivo is open to debate (see below). The filter itself is also a physical barrier that recruited cells must traverse and detach from in order to enter the stromal environment. Indeed, it takes considerably longer for lymphocytes to move through the filters than it would to move through the basement membrane and into tissue in vivo (Tsuzuki et al., 1996; Westermann et al., 1997) or across EC monolayers in vitro (McGettrick et al., 2009a), i.e., hours as opposed to minutes. It is also notable that in certain circumstances we observe little effect of cytokine-treatment on adhesion. It is well known that prolonged incubation (~ hours) significantly augments leukocyte adhesion independent of the activation status of the endothelium (e.g. Oppenheimer-Marks et al., 1991; McGettrick et al., 2009a). However, specificity of cytokine-induced effects can be greatly improved by reducing the settling period to minutes or introducing flow, indicating that cytokine treatment is more important for the initial recruitment phase than the onward migration of leukocytes.

To investigate some of the limitations noted above, we incorporated fibroblasts in collagen gels and either cultured EC directly on top or on filters placed above the gels. Extracellular matrix gels of this type are common substrates used to study migration of cells in 3-D, including lymphocytes (e.g. Friedl et al., 1995; Wolf et al., 2009). In addition, transendothelial migration of T-cells has been studied for EC grown on collagen gels, where only ~ 10% migrated into the gel (Pietschmann et al., 1992; Brezinschek et al., 1995). However, studies of EC on gels incorporating fibroblasts have not been reported previously. Perhaps surprisingly, we found that fibroblasts reduced PBL migration through EC seeded directly on the gel, but not through EC cultured on filters placed above the gels. However, we also noted that on some occasions EC monolayers had poor integrity when formed directly on the surface of fibroblast gels. Others have found that direct EC–fibroblast contact can trigger EC to migrate and form tube-like structures (Sorrell et al., 2008), but we did not observe this on the gels. However, we have reported previously that close EC–FB contact (on either side of 3 μm-pore filters) ablated lymphocyte capture from flow, most likely by altering the ability of EC to present chemokines (McGettrick et al., 2010). In contrast, smooth muscle cells (SMC) incorporated into collagen gels were reported to promote mononuclear leukocyte migration across EC overlaying the gel (Chen et al., 2006; Navab et al., 1988). In vivo, EC grow on a basement membrane in close juxtaposition with pericytes or smooth muscle cells depending on the vessel, but may not usually have direct contact with fibroblasts. Here, behaviour (morphology, recruitment of leukocytes and response to cytokines) of EC was not impaired when cultured on collagen matrix alone or as part of the double gel model (where EC are seeded above a gel, above a fibroblast containing gel). Such behaviour is similar to that observed when endothelial cells are cultured on a range of surfaces, including plastic tissue culture wells and Transwell filters (McGettrick et al., 2009a). This indicates that collagen itself is unlikely to impair EC function or behaviour, rather the loss of integrity was a fibroblast-specific effect. The integrity of the endothelium may be differentially modulated by different stromal cells in vitro, and so the best model for co-culture might be different also. These findings do, however, raise the question as to whether fibroblasts potentiated lymphocyte transmigration at the level of the filter rather than the endothelium in that model. This was investigated further in the layered-gel model (see below).

In either model, fibroblasts reduced the proportion of transmigrated PBL that penetrated into the gel and those that did enter migrated only half as deep when fibroblasts were present. Of note, responses to cytokine-treatment were similar for fibroblasts cultured on plastic as those within the gel. In fact, higher levels of the adhesion molecules, ICAM-1, were observed in co-culture gel constructs, indicating that the fibroblasts had sufficient receptors to support and encourage lymphocyte migration through the gel. Density, spatial arrangement and source of collagen fibres have all been suggested to alter the ability of leukocytes to move within gel constructs (Wolf et al., 2009). Here it became evident that fibroblasts caused significant contraction and reduction in depth of the gels. When we purposely made gels with increasing collagen concentrations, the inhibition of initial penetration was reproduced. Thus the main effect of the fibroblasts in the later stages of migration appeared to be through matrix modification, while effects through direct contact with the PBL or release of attractants were not obvious. The fibroblasts probably also deposited matrix proteins such as fibronectin over the duration of the culture and assay, and it would be interesting to investigate whether this might affect migration in the future. Preliminary studies where we have purposely added fibronectin into the collagen gels did not, however, cause increased penetration at least (G. Jevons; unpublished observations). Others have reduced fibroblast contraction of collagen gels through the chelation of divalent cations (e.g. Ca^2 +^) (Ilagan et al., 2010) or the antagonism of endogenous TGFβ signalling or heparin sulfate-containing proteoglycan synthesis (Chen et al., 2005). However, such interventions would have direct effects on the recruitment process itself, and thus be inappropriate to use in models of lymphocyte migration.

Following from the issues raised by direct contact between EC and fibroblasts, barrier effects of filters and gel contraction, we developed a ‘double gel’ model. This provided a contiguous system of cells and tissue-like matrix that might be more physiologically relevant than our alternative models. Under these conditions, fibroblasts enhanced the numbers of lymphocytes migrating through the EC, but had no effect on their subsequent migration potential through the gel. Thus, the results supported the conclusion that fibroblasts promoted transendothelial migration of PBL through remote effects of soluble mediators but influenced penetration of tissue mainly by modifying matrix structure. Few PBL reached the fibroblast zone after 24 h in this construct, and it would be necessary to either reduce the thickness of the upper gel layer or extend the duration of the assay, to test whether fibroblasts could influence motility of lymphocytes by direct contact.

In all of the models, ability to retrieve cells that have migrated into the different regions allowed us to study differential responses of lymphocyte subsets without costly and potentially property-changing pre-isolation procedures. Using immuno-labelling and flow cytometry, we were able to show that T-cells (CD4 and CD8) and B-cells migrated across endothelial mono and co-cultures with equal ability in the two models examined (multi-filter and filter-gel). Moreover, effector memory T-cells showed an enhanced migratory capacity, preferentially migrating through EC. Thus the process of transendothelial migration does not appear to be selective at the level of T- and B-cells, but could potentially select for discrete subpopulations such as effector memory. Interestingly, migration of T-cells, but not B-cells, into matrix or through the stromal-filter layer was adversely affected by the presence of fibroblasts. In light of the above, these findings suggest that it is the migration potential of T-cells that is sensitive to modifications in the matrix structure. It is possible that B-cells may be better able to remodel the matrix to create pathways for their entry making them less sensitive to structural changes within the matrix. An alternative explanation is that fibroblast-derived mediators are more attractive to B-cells than T-cells. For example, it has been reported that B-cells adhere more efficiently to human dermal fibroblasts than T-cells (Couture et al., 2009). Moreover, B-cells, but not T-cells, were able to migrate through a fibroblast barrier (monolayer) (Couture et al., 2009). In fact in that study the fibroblasts appeared to selectively promote B-cell migration. Our understanding of comparative lymphocyte (T-cell vs. B-cell) migration through tissue matrix during inflammation is limited and requires further investigation.

The models we describe could be adapted to incorporate stromal cells from other tissues or those associated with specific disorders. This might enable investigation of tissue-specific regulatory pathways acting at the endothelial or leukocyte level. Alternatively, disruption of normal processes in a range of inflammatory conditions and cancers might be studied. We have already shown that transformed fibroblasts from joints with rheumatoid arthritis can induce initial adhesion of flowing leukocytes (Lally et al., 2005; McGettrick et al., 2009b), and are now using the models described here to test whether subsequent behaviour is also modified. Potential therapeutic agents which target diseased stromal cells, or the abnormal pathways they initiate, to restore normal patterns of lymphocyte recruitment, could also be screened in our models. Based on the above, the model chosen may vary depending on the stromal cell under investigation and its expected proximity to EC or effect on matrix structure. While the model with EC cultured above a double-layered gel with stromal cells held remote may be the most appropriate for studying effects of fibroblasts, this might not be the case for cells more typically in close contact with EC, or where changes in matrix properties are of specific interest.
