# Supplementary material for: Analysis of the effects of stromal cells on the migration of lymphocytes into and through inflamed tissue using 3-D culture models
Source: J Immunol Methods. 2013 Dec 31;400-401:45–57. doi: 10.1016/j.jim.2013.10.004 (PMC3878567; doi:10.1016/j.jim.2013.10.004)
Supplement: Supplementary file 1 — Supplementary material. [file mmc1.docx]

**Supplemental Figure Legends**

**Supplemental Figure 1:** **Gating strategy for the analysis of lymphocytes by flow cytometry.** Initial PBL or PBL harvested from compartments of the 3D tissue constructs were surface stained with antibodies against the pan T cell (CD3) and B cell (CD19) markers, the major T-cell subset markers (CD4 and CD8) and antigens that define T-cell memory phenotype (CD45Ra and CD62L). **(A)** Gated PBL population was identified based on forward scatter v side scatter. Cell doublets were excluded based on pulse width. **(B)** Using the PBL gate, CD19 positive B-cells and CD3 positive T-cells were then defined. **(C)** Using the CD3 gate, CD4 positive and CD8 positive cells were defined. **(D)** T-cells were further defined as based on expression of CD62L and CD45RA to attribute: Naïve (N): CD45Ra+/CD62L+; Central Memory (CM): CD45Ra-/CD62L+; Effector Memory (EM): CD45Ra-/CD62L-.

**Supplemental Figure 2: Expression of adhesion receptors and chemokine ligands by endothelial cells cultured on filters.** Endothelial cells were cultured on 3.0um pore filters for 24h prior to treatment with (filled bars) or without (open bars) TNF+IFN. Surface expression of **(A)** E-selectin and **(B)** VCAM-1 were determined by flow cytometry. **(C)** Gene expression of the CXCR3 ligands, CXCL9-11, were determined by RT-PCR and expressed as a percentage of the β-actin band. Data are mean ± SEM from 3 experiments. * =p<0.05 and ** = p<0.01 compared to untreated EC control (none) by paired t-test.

**Supplemental Figure 3**: **Loss of endothelium from the surfaces of co-culture gels.** The endothelium coated surfaces of single thickness mono-culture (blank) **(A)** and co-culture (fibroblast-containing) **(B)** collagen type 1 gels after the removal of non adherent PBL by washing.

**Supplemental Figure 4: Expression of adhesion receptors and chemokines by fibroblasts cultured in gels.** Fibroblasts were cultured on plastic (2D), incorporated into a gel alone (3D) or with EC (3D co-culture) for 24h, prior to treatment with (filled bars) or without (open bars) TNF+IFN. **(A)** Surface expression of ICAM-1 was determined by flow cytometry. ANOVA shows a significant effect of cytokine treatment on ICAM-1 expression, p<0.001. **(B)** Secretion of CXCL1 and CXCL10 was determined by Luminex and expressed as pg/ml. Data are mean ± SEM from 3 experiments. ND = not done. ** = p<0.01 compared to untreated control by Bonferroni test.

**Supplemental Figure 1
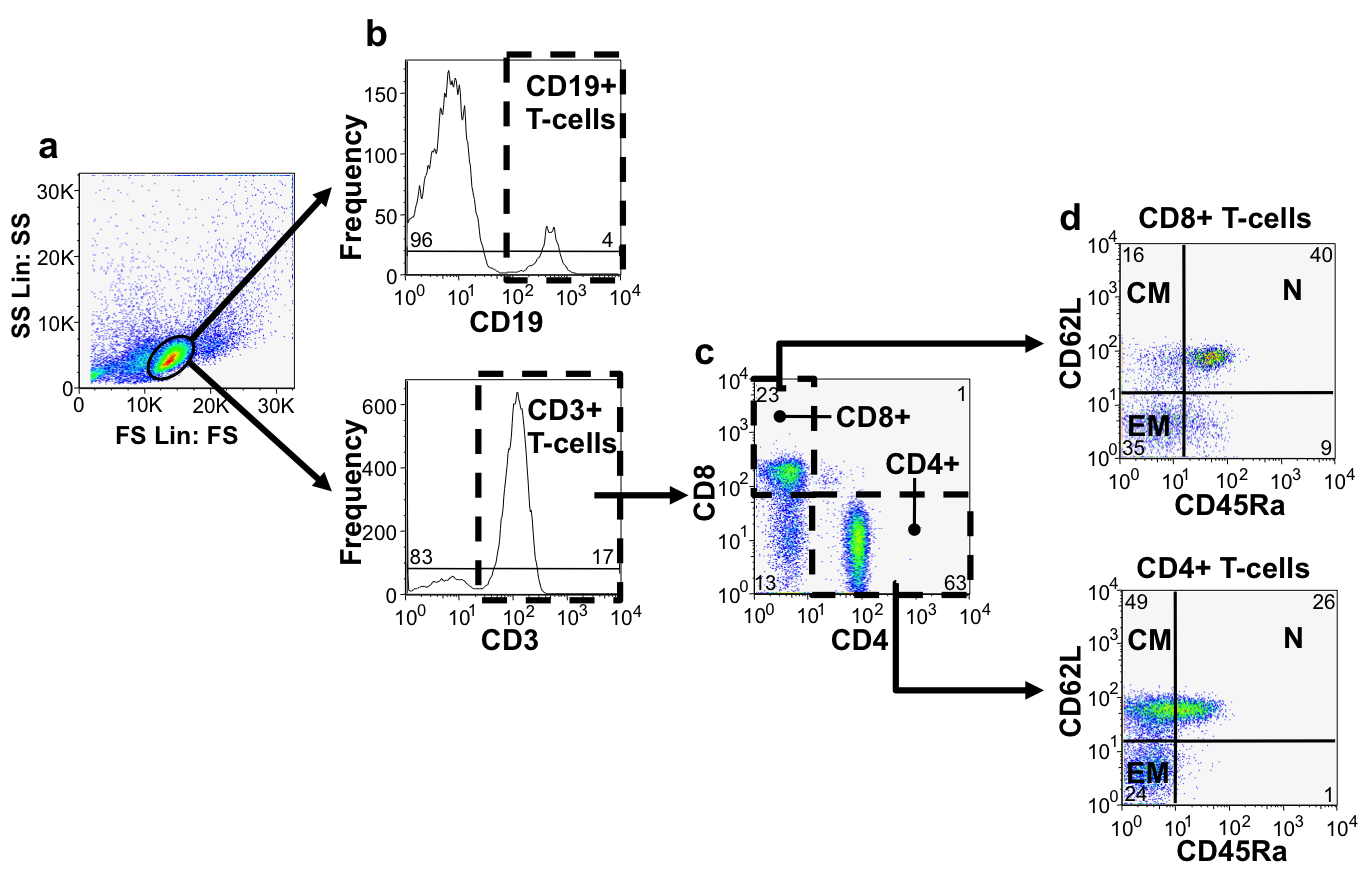
**

**Supplemental Figure 2**

**Supplemental Figure 3**

**
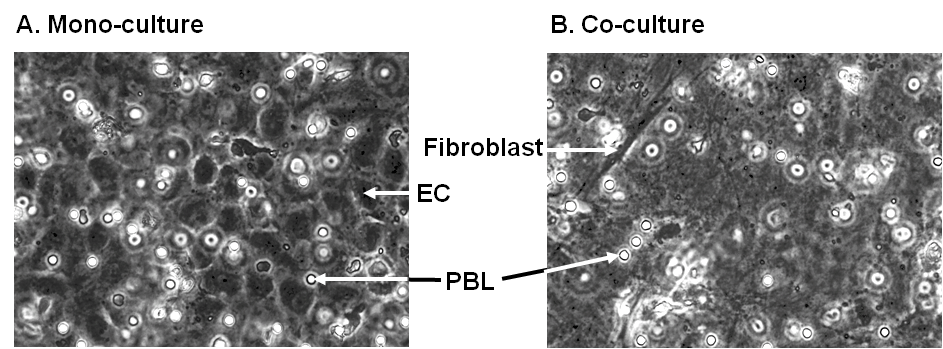
**

**Supplemental Figure 4**
